# A mega-analysis of fixed-dose trials reveals dose-dependency and a rapid onset of action for the antidepressant effect of three selective serotonin reuptake inhibitors

**DOI:** 10.1038/tp.2016.104

**Published:** 2016-06-07

**Authors:** F Hieronymus, S Nilsson, E Eriksson

**Affiliations:** 1 Department of Pharmacology, Sahlgrenska Academy, University of Gothenburg, Gothenburg, Sweden; 2 Institute of Mathematical Sciences, Chalmers University of Technology, Gothenburg, Sweden

## Abstract

The possible dose-dependency for the antidepressant effect of selective serotonin reuptake inhibitors (SSRIs) remains controversial. We believe we have conducted the first comprehensive patient-level mega-analysis exploring this issue, one incentive being to address the possibility that inclusion of low-dose arms in previous meta-analyses may have caused an underestimation of the efficacy of these drugs. All company-sponsored, acute-phase, placebo-controlled, fixed-dose trials using the Hamilton Depression Rating Scale (HDRS) and conducted to evaluate the effect of citalopram, paroxetine or sertraline in adult major depression were included (11 trials, *n*=2859 patients). The single-item depressed mood, which has proven a more sensitive measure to detect an antidepressant signal than the sum score of all HDRS items, was designated the primary effect parameter. Doses below or at the lower end of the usually recommended dose range (citalopram: 10–20 mg, paroxetine: 10 mg; sertraline: 50 mg) were superior to placebo but inferior to higher doses, hence confirming a dose-dependency to be at hand. In contrast, among doses above these, there was no indication of a dose–response relationship. The effect size (ES) after exclusion of suboptimal doses was of a more respectable magnitude (0.5) than that usually attributed to the antidepressant effect of the SSRIs. In conclusion, the observation that low doses are less effective than higher ones challenges the oft-cited view that the effect of the SSRIs is not dose-dependent and hence not caused by a specific, pharmacological antidepressant action. Moreover, we suggest that inclusion of suboptimal doses in previous meta-analyses has led to an underestimation of the efficacy of these drugs.

## Introduction

Although selective serotonin reuptake inhibitors (SSRIs) have since long been extensively used for the treatment of depression, it remains controversial whether they display a dose–response relationship. Thus, while a number of individual studies and meta-analyses do suggest a dose-dependency to be at hand,^1, 2, 3, 4, 5, 6, 7^ several authors systematically reviewing the outcomes of individual studies have concluded that high doses are no more effective than low,^1, 2, 3^ the dose–response curve often being described as flat. This possible lack of a dose–response relationship for the SSRIs has recently gained renewed interest, as it has been put forward as an argument for the claim that they are devoid of specific, pharmacological antidepressant actions.^8, 9, 10^ To clarify whether the antidepressant effect of the SSRIs is indeed unrelated to dose has, for this reason, emerged as a task of considerable theoretical importance.

This matter has obvious bearing on clinical practice. If high doses are no more effective than the lowest recommended one, the commonly applied dose escalation strategy in cases of non-response may be counterproductive by needlessly increasing the burden of side effects. In addition, if the effect of an SSRI is an all-or-nothing phenomenon, the SSRIs should perhaps preferably be prescribed at doses even lower than those currently recommended. On the other hand, if these drugs do display dose-dependency, the widespread assumption that they do not may prompt clinicians to use suboptimal dosage.^11^ Illustrating the unfortunate ambiguity of the current evidence on this matter, two British guidelines state that no dose-dependency has been established,^4, 5^ whereas the American Psychiatric Association recommends dose escalation in cases of non-response.^6^

The effect sizes (ES) for comparisons of SSRIs versus placebo in drug company-sponsored trials using the conventional measure of efficacy are usually small (0.3–0.4).^12^ Regardless of whether such modest outcomes are true reflections of a relatively poor efficacy,^8, 13, 14^ or whether they are artificially low because of the methodological shortcomings marring company-sponsored trials,^15, 16, 17^ they justify the conclusion that detecting significant differences between two doses that are both superior to placebo would require a large number of subjects.^3, 15^ Most individual dose–response studies being marred by too low statistical power to detect differences between doses might, hence, partly explain why previous systematic reviews have reached the conclusion that no dose–response relationship is at hand.^1, 2, 3^ Supporting this possibility, three^7, 18, 19^ out of four^8^ meta-analyses addressing this issue do, in fact, suggest the antidepressant effect of the SSRIs to be dose-dependent.

One aspect that may have hampered the attempts to reveal differences between doses in SSRI trials is the common use of the reduction in the sum rating of the 17 items on the Hamilton Depression Rating Scale (*HDRS-17-sum*) as effect parameter.^20^ As some of the symptoms included in this multidimensional inventory are absent in many depressed patients already at baseline, and some are common also in non-depressed subjects and/or are frequent side effects of SSRIs, the feasibility of this measure for detecting differences between groups in SSRI trials has been frequently questioned.^16, 17, 20, 21^ Accordingly, we recently showed the likelihood of detecting an antidepressant signal in placebo-controlled trials to be far higher when using one key item from this scale, *depressed mood* (rated 0–4), rather than *HDRS-17-sum*, as a measure.^17^

In order to clarify the question of a possible dose-dependency for the SSRIs across the entire possible dose range, that is, from doses lower than those recommended to the highest that have been tested in fixed-dose studies, we have conducted a *post hoc* patient-level mega-analysis using *depressed mood* as primary and *HDRS-17-sum* as secondary effect parameters. To avoid any bias in the selection of studies, and to obtain sufficient statistical power, all company-sponsored fixed-dose, placebo-controlled trials conducted for three of the first-generation SSRIs, citalopram, paroxetine and sertraline, were included.

## Materials and methods

### Data acquisition

Patient-level data from all drug company-sponsored fixed-dose, placebo-controlled trials regarding the treatment of depression in adults that have been conducted for citalopram, escitalopram, paroxetine, fluoxetine and sertraline were requested from GlaxoSmithKline (GSK, Brentford, UK; paroxetine), Lilly (Indianapolis, IN, USA; fluoxetine), Lundbeck (Valby, Denmark; citalopram and escitalopram), Actavis (Parsippany-Troy Hills, NJ, USA; escitalopram) and Pfizer (New York, NY, USA; sertraline). Data for fluvoxamine were not requested, as it was deemed sufficient to include those SSRIs that are currently commonly prescribed for the treatment of depression. Whereas the manufacturers of citalopram, paroxetine and sertraline sent us the requested data, individual item data on fluoxetine were unfortunately not available in an electronic format and could hence not be delivered. Although we did obtain patient-level data also from the escitalopram trials conducted by Lundbeck, these were not relevant for this analysis since only one dose (10 mg) had been tested using a fixed-dose design. We have tried to obtain patient-level data from trials sponsored by the other company involved in the clinical trial program for escitalopram, that is, Forest, but the company that has since then acquired Forest, that is, Actavis, has unfortunately not been able to submit the requested information in electronic format. We confirmed that we had access to all pertinent studies regarding citalopram, paroxetine and sertraline by examining the Food and Drug Administration-approval packages for the relevant drugs.^17^

Only two sertraline studies (PZ/101 and PZ/310) comprised a 400-mg dose, and these lasted for 4 weeks only; hence, no efficacy measures were available for this dosage beyond week 4. The polymeric matrix used in paroxetine-controlled-release (CR) releases ~80% of the active compound;^22^ the company hence used the doses 12.5 and 25 mg of paroxetine CR, assuming that they should correspond to 10 and 20 mg of paroxetine immediate-release. We have similarly assumed that patients medicating with paroxetine CR 12.5 or 25 mg per day received a daily paroxetine dose of 10 and 20 mg, respectively. None of the studies included a comparator SSRI given in a fixed-dose manner; therefore, for each study only data regarding one compound were obtained.

### Statistics

Recently, mixed models for repeated measurements have replaced last-observation-carried-forward-based analysis of covariance as the preferred methodology for antidepressant trials.^23^ For all analyses on ordinal outcome measures, we, hence, implemented a linear mixed model using the PROC MIXED procedure in SAS version 9.4 (SAS Institute, Cary, NC, USA). The basic model included change score for the relevant measure (*depressed mood* or *HDRS-17-sum*) as the dependent variable, time (*week*) and trial as fixed factors, and baseline rating on the outcome parameter as covariate. An unstructured (co)variance structure was used to model within-patient errors. Denominator degrees of freedom were estimated using the Kenward–Roger approximation. ES (Cohen's *d*) were calculated by dividing the least-squares means differences for the relevant contrast by the root of the variance for the corresponding time point taken from the covariance matrix. For analyses spanning multiple weeks, the between-treatment contrast(s) at the last evaluation (usually week 6) were considered primary outcome. *P*-values are reported without adjustment for multiple comparisons; all significance tests reported are two-sided. Whereas the *depressed mood* item of the Hamilton Depression Rating Scale was regarded as the primary effect parameter, most analyses were repeated using *HDRS-17-sum* as an alternative measure.

To shed light on the possible relationship between dose and effect for each antidepressant, we first modeled dose as a categorical predictor for all drugs separately and including only the placebo cases from the trials in which the drug in question had been evaluated. Here the basic model was extended by including a fixed factor for dose and the interaction between dose and time. For these analyses, only *depressed mood* was used as effect parameter.

We then conducted a pooled dose–response analysis comprising all three SSRIs and using both *depressed mood* and *HDRS-17-sum* as effect parameters. As the issue of dose-equivalency between different SSRIs remains unsettled,^18^ visual inspection of the results of the drug-specific analyses was used to produce an optimal-dose and a low-dose group. The basic model was extended by including a fixed factor for dose group (placebo, low-dose and optimal-dose) and the interaction between dose group and time. Acknowledging the exploratory nature of the pooling procedure, we also re-ran this analysis including only the lowest and highest doses for each compound. For sertraline, the 200-mg dose was used for this purpose as the highest dose (400 mg) had been evaluated in 4-week studies only.

We also modeled dose as a linear covariate for all SSRI cases pooled, but excluding placebo-treated patients, using both *depressed mood* and *HDRS-17-sum* as outcome measures; here the interaction between time and the linearized dose covariate was added to the basic model. Doses were normalized so that the lowest dose for each drug was anchored at zero and the highest at one, doses in between being linear interpolations between these two.

As the pooled assessment comprising all three SSRIs was focused on the possible difference between doses at the lower end of the dose–response curves on the one hand and all higher doses on the other, but not within the dose range that may be described as medium to high, we also conducted a pooled analysis where only doses within the latter range (that is, citalopram ⩾40 mg, paroxetine ⩾20 mg and sertraline ⩾100 mg) were included. Again, dose was treated as a linear covariate and normalized so that the lowest included dose was anchored at zero and the highest at one, doses in between being linear interpolations between these two.

Finally, we modeled the likelihood of achieving response or remission in the pooled population again divided into the low-dose and optimal-dose groups. For *depressed mood*, response was defined as a lower rating than at baseline and remission as a rating of zero. For *HDRS-17-sum*, response was defined as a ⩾50% decrease and remission as a score of ⩽7.^4, 5, 6^ The models were fitted using the PROC GLIMMIX procedure and included time (*week*), dose group, trial and the interaction between time and dose group as fixed factors, and the baseline ratings of *depressed mood* or *HDRS-17-sum*, respectively, as covariate. All time points were included in the model but only the week 6 results are reported. The model used a binary distribution with a logit link, the Kenward–Roger approximation was applied to estimate denominator degrees of freedom and an unstructured (co)variance structure was used to model the within-patient errors.

### Ethics

The Regional Ethical Review Board reviewed the study protocol and issued an advisory opinion stating no objection.

## Results

The data set consists of 2859 patients with valid baseline data and at least one post-baseline measurement (Table 1). Of these, 600 received citalopram (10–60 mg), 1043 paroxetine (10–40 mg), 481 sertraline (50–400 mg) and 735 placebo.

**Figure 1:**
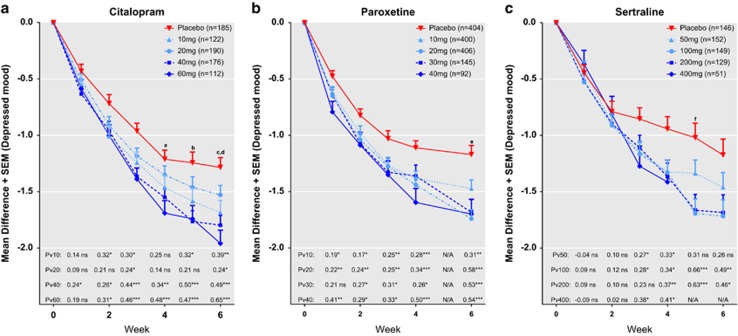
Trajectories of change, effect sizes (ES) and levels of significance for individual drugs (**a**, citalopram; **b**, paroxetine; **c**, sertraline) and doses. Shown are the mean reductions in *depressed mood*. ES and levels of significance for all comparisons versus placebo (P) are displayed in the lower parts of the graph: *P<0.05, **P<0.01, ***P<0.001. Between-dose differences that are statistically significant are indicated with the superscripted letters a–f. ^a^60 and 20 mg citalopram (ES: 0.34, *P*=0.01), ^b^40 and 20 mg citalopram (ES: 0.29, *P*=0.02), ^c^40 and 20 mg citalopram (ES: 0.26, *P*=0.03), ^d^60 and 20 mg citalopram (ES: 0.41, *P*=0.003), ^e^20 and 10 mg paroxetine (ES: 0.27, *P*=0.005) and ^f^100 and 50 mg sertraline (ES: 0.35, *P*=0.04).

Figure 1 shows mean changes, effect sizes and levels of significance for all individual doses of the three drugs when using change in *depressed mood* as outcome parameter. In the paroxetine studies, no evaluations had been made at week 5. All drugs and doses showed significant superiority over placebo at some point of observation; of particular note is that citalopram (40 mg) and paroxetine (10, 20 and 40 mg) separated significantly from placebo already after 1 week. Differences between doses only occasionally reached statistical significance.

Inspection of the dose curves for citalopram suggests the effect of 10 mg to be similar to that of 20 mg, but both these doses display lower efficacy than 40 and 60 mg; for the pooled analysis, 10 and 20 mg were, hence, classified as low doses, whereas 40 and 60 mg were regarded as optimal doses. For paroxetine, the largest difference was seen between the 10 mg group on the one hand and the 20, 30 and 40 mg groups on the other; hence, 10 mg was the only dose classified as low. For sertraline there was no apparent difference between 100 , 200 and 400 mg, whereas 50 mg produced a lower response; consequently, 50 mg was designated as low and the other doses as optimal.

**Figure 2:**
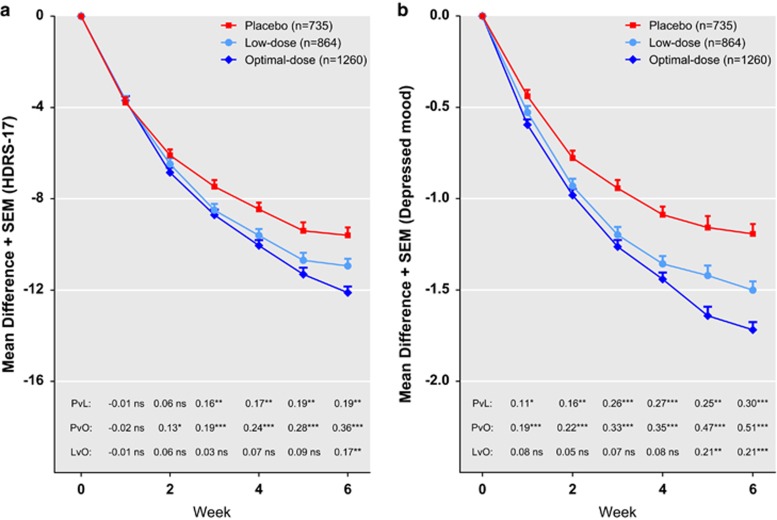
Trajectories of change, effect sizes (ES) and levels of significance for the pooled analyses comprising all subjects. Shown are the mean reductions on the 17 item Hamilton Depression Rating Scale (*HDRS-17-sum*) (panel **a**) and *depressed mood* (panel **b**). ES and levels of significance for all comparisons between treatment groups are displayed in the lower parts of the graph (L, low-dose; O, optimal-dose; P, placebo; v, versus): *P<0.05, **P<0.01, ***P<0.001.

Figure 2 displays levels of significance, ES and the mean changes in *HDRS-17-sum* (Figure 2a) and *depressed mood* (Figure 2b) for the pooled dose groups. With respect to reduction in *depressed mood*, both dose groups outperformed placebo after 1 week, optimal-dose outperforming low-dose after 5 weeks. With respect to reduction in *HDRS-17-sum*, both doses separated significantly from placebo after 2–3 weeks of treatment, optimal-dose outperforming low-dose after 6 weeks.

**Figure 3:**
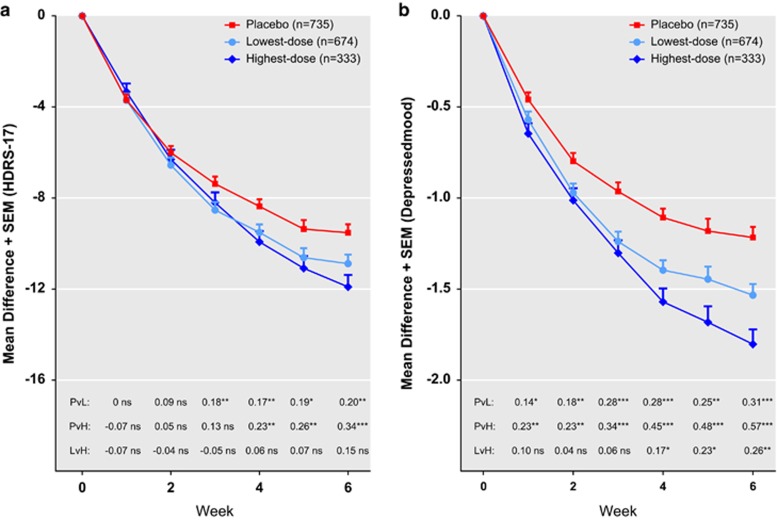
Trajectories of change, effect sizes (ES) and levels of significance for the sensitivity analyses (lowest versus highest doses). Shown are the mean reductions on the 17 item Hamilton Depression Rating Scale (*HDRS-17-sum*) (panel **a**) and *depressed mood* (panel **b**). ES and levels of significance for all comparisons between treatment groups are displayed in the lower parts of the graph (H, highest dose; L, lowest dose; P, placebo; v, versus): *P<0.05, **P<0.01, ***P<0.001.

Figure 3 is equivalent to Figure 2 but displays the results of the sensitivity analysis including only the lowest and highest doses for each compound. With respect to reduction in *HDRS-17-sum*, both dose groups separated significantly from placebo after 3–4 weeks of treatment; however, the difference between the lowest and the highest doses was never significant (week 6: *P*=0.08). With respect to reduction in *depressed mood*, both dose groups separated significantly from placebo after 1 week and from each other after 4 weeks and onwards.

For the overall linearized dose analysis using *depressed mood* as the effect parameter, a significant effect of dose was found at week 3 (*β*=−0.15 (0.08), *t*=−2.05, *P*=0.04), 4 (*β*=−0.21 (0.08), *t*=−2.67, *P*=0.008), 5 (*β*=−0.25 (0.10), *t*=−2.43, *P*=0.02) and 6 (*β*=−0.31 (0.09), *t*=−3.55, *P*<0.001). When *HDRS-17-sum* was instead used as the measure of response, a significant dose effect was observed at week 6 (*β*=−1.14 (0.57), *t*=−1.98, *P*=0.047). For the subgroup analysis within the optimal-dose group there was, on the other hand, no significant effect of dose at any point of assessment either with respect to *depressed mood* (week 6: *β*=−0.09 (0.15), *t*=−0.64, *P*=0.5) or with respect to *HDRS-17-sum* (week 6: *β*=1.05 (0.96), *t*=1.10, *P*=0.3).

Table 2 details the analyses of categorical outcomes at week 6. Optimal-dose was significantly superior to low-dose both with respect to the percentage of subjects showing response using the *depressed mood* item and with respect to the percentage of subjects displaying no *depressed mood* at week 6. Similar results were found using *HDRS-17-sum* as the measure. For all measures, both low-dose and optimal-dose SSRI outperformed placebo.

## Discussion

This study differs from previous attempts to disclose a possible dose–response relationship for the antidepressant effect of SSRIs in three regards: by including a more sensitive effect parameter (the *depressed mood* item) than the usual one (*HDRS-17-sum*),^17^ by using a statistical method (mixed models for repeated measurement) more appropriate for handling missing data than previous techniques^23^ and by being based on patient-level data from all relevant trials of the three study drugs, the latter premise precluding the influence of publication bias and rendering the studied population unusually large for a patient-based analysis. As a result, we made three observations, partly challenging the conventional wisdom. First, we could refute the assumption of a flat dose–response curve; thus, whereas doses below or at the lower end of the dose range usually recommended were superior to placebo, they were inferior to higher doses. Second, the ES for the difference between optimal doses of SSRIs and placebo with respect to reduction in *depressed mood* was considerably larger than that usually attributed to these drugs. And third, SSRIs outperformed placebo already after 1 week of treatment.

In spite of the fact that antidepressant trials are marred by methodological shortcomings that we could not adjust for in this analysis, and most of which are likely to make the difference between two treatments, appear smaller than it actually is;^15, 16, 17^ the ES for the difference between placebo and optimal-dose SSRI with respect to reduction in *depressed mood* was 0.5–0.6 (Figures 2 and 3), that is, well on par with that reported for many well-established treatments in somatic medicine.^24^ We suggest that previous assessments of the usefulness of the SSRIs^8^ have understated their efficacy, both by being based on a questionable measure of response and also by including patients treated with suboptimal doses. In addition, whereas the alleged lack of a dose–response relationship for the SSRIs has been used as an argument for the claim that these drugs are devoid of specific antidepressant properties,^8, 9, 10^ the finding that optimal doses are indeed superior to lower though effective ones refutes this assumption.

As many different symptoms contribute to the distress associated with depression, important aspects of a given treatment may be overlooked when measuring severity by assessing one symptom only; regulatory authorities hence have required the use of multi-item scales, such as the HDRS, for evaluating efficacy. In addition, supporting this stance, it has been demonstrated that single-item measures usually result in a lower inter-rating reliability than multi-item rating scales.^20^ On the other hand, the use of a measure of efficacy composed by the sum score of 17 symptoms, many of which are often not present at baseline, and many of which may be present at end point also in patients who have recovered, is bound to result in suboptimal sensitivity by enhancing variability, hence making the tested compound appear less effective than it actually is. In line with this, we found differences between doses (and between active medication and placebo) to be larger when replacing the conventional effect parameter, that is, the *HDRS-17-sum*, with a single symptom that is present in most subjects at baseline and of obvious clinical relevance, that is, *depressed mood*. This observation is well in line with our previous report (partly based on the same data set) showing that the *depressed mood* item from the HDRS is a considerably more sensitive measure than the *HDRS-17-sum* for detecting an antidepressant signal.^17^ The pros and cons of single-item assessments versus multi-item assessments have been elaborated upon elsewhere.^17, 20, 21^

The ES at end point for the differences between optimal-dose and low-dose SSRIs were 0.21 (*depressed mood*) and 0.17 (*HDRS-17-sum*), respectively. ES of these moderate magnitudes for the influence of dose are compatible with the assumption that the discrepancy between those previous meta-analyses that have obtained support for a dose–response relationship^7, 18, 19^ and the systematic reviews that have not^1, 2, 3^ may partly be a matter of statistical power.

A superiority of optimal-dose over low-dose could be explained either by a lower number of non-responders among those given an optimal dose or by a larger symptom reduction in responders given an optimal dose than in responders given a low dose. The analyses of categorical outcomes suggest both alternatives to be relevant; optimal doses hence were associated both with fewer subjects not reporting any reduction in *depressed mood* and with more patients reporting no *depressed mood* at the week 6 end point (Table 2). Of note, and again underlining the benefit of SSRIs, is that more than 1/3 of patients in the optimal-dose group, but less than 1/6 of those on placebo, reported absence of *depressed mood* at the week 6 end point.

Beyond the conclusion that doses of SSRIs below or at the lower end of the dose range usually recommended are inferior to higher doses, we caution against using our results for dosing recommendations, the number of patients given each dose of each compound being relatively low. In addition, it should be noted that the proportion of subjects given high and low doses differ between drugs; subjects given high doses of paroxetine were thus relatively few. Moreover, with respect to sertraline, only one of the three trials including the 50 mg dose lasted for 6 weeks. Taking these caveats into consideration, inspection of the data regarding the individual drugs (Figure 1), however, suggests that the effect of sertraline is likely to be augmented by dose escalation from the lowest recommended dose (50 mg), which is at odds with some previous reports.^25, 26^ With respect to citalopram, a dose of 20 mg, which is a recommended dose in some European countries, such as the United Kingdom, but lower than the lowest recommended dose in the United States (40 mg), appeared less effective than doses of 40 mg and above; of note is however that citalopram doses higher than 40 mg should be avoided because of a possible risk for QTc prolongation.^27^ Paroxetine seems to display its maximum efficacy at the minimal recommended dose (20 mg), which is in line with a report suggesting that higher doses of this compound cause no further increase in serotonin transporter occupancy.^28^

Whereas the pooled analyses revealed clear-cut differences between low (yet not ineffective) doses and higher ones, inspection of the dose–response curves for the individual drugs suggests these to plateau at 20 mg for paroxetine, 40 mg for citalopram and 100 mg for sertraline. These doses are all within the ranges generally recommended for the treatment of depression, e.g., by the FDA (sertraline: 50–200 mg, citalopram: 40 mg, paroxetine: 20-50 mg); however, for citalopram and sertraline, but not for paroxetine, they are higher than the World Health Organization's defined daily dose for moderately severe depression (sertraline: 50 mg, citalopram: 20 mg, paroxetine: 20 mg).

The assumption that doses above 20 mg for paroxetine, 40 mg for citalopram and 100 mg for sertraline are not associated with a further increase in antidepressant effect gains support from the fact that the linear analysis conducted within the medium-to-high dose range yielded no significant effect of dose. On the other hand, these results contrast to those of a recent meta-analysis suggesting very high doses (sertraline: 240–300 mg, citalopram: 60–75 mg, paroxetine: 40–50 mg) to be superior to medium ones.^7^ Whereas our mega-analysis is based on fixed-dose studies only, this meta-analysis was, to a considerable extent, based on flexible-dose studies that appear to have been categorized as if all patients had actually been given the highest dose allowed; as the reported median dose in flexible-dose studies usually is considerably lower than the maximal dose, this assumption may however be questioned. Further, as the highest doses allowed in flexible-dose studies are often higher than the highest doses tested in fixed-dose studies, it is not unlikely that the apparent superiority of very high doses suggested by Jakubovski and co-workers,^29^ doses that have rarely been tested in fixed-dose studies, might in fact be an effect of study design (flexible dosage) rather than of the dose itself. Other differences between the two studies are that the present analysis is based on patient-level data and, for the three studied drugs, comprises all company-sponsored trials, including the non-published ones, hence avoiding the risk of publication bias.

The observation of a significant difference between active drug and placebo in reducing *depressed mood* (but not *HDRS-17-sum*) as early as after 1 week is in line with previous data regarding another SSRI, fluvoxamine,^30^ but contrasts to the common view that SSRIs require 2 weeks or more to exert any antidepressant effect.^31^ Although the symptom reduction observed after 1 week is small, and of questionable clinical relevance, it is of considerable theoretical interest as it casts doubt on the assumption that a likely mechanism of action of the SSRIs is one that requires prolonged administration, such as, for example, the formation of new nerve cells.^31^ Instead, our data suggest the antidepressant effect to be the result of a process that, although gradual and sluggish, is manifested already within the first week of treatment, a view gaining support also by studies addressing the influence of SSRIs on emotional processing.^32^ In this context, it should be noted that there are, in fact, a number of previous studies suggesting SSRIs to outperform placebo within a week also with respect to reducing *HDRS-17-sum*.^33, 34^

This study has certain limitations. One such is that not all SSRIs were included for reasons presented above. This drawback, however, does not preclude the major conclusions of the study, that is, that the antidepressant effect of SSRIs, in contrast to what has been claimed by others, may display a dose–response relationship, and that at least some SSRIs outperform placebo already after 1 week of treatment. A second limitation is that we had no possibility to relate serum levels of the given compound with response as such analyses unfortunately are seldom included in this kind of company-sponsored trials. It should also be noted that the fixed-dose design of the included trials does not allow for any definite conclusion regarding the possible benefit of increasing the dose in a subgroup of patients who have failed to respond to a given dose. Finally, for the individual patient, the possible benefit of giving a higher dose must, needless to say, be weighed against the possible drawback of a greater side effect burden; while the aim of this study was to shed light on the possible association between dose and efficacy, our analyses do not provide any information regarding the tolerability of higher doses as compared to lower.

To summarize, the present study permits three conclusions. First, the antidepressant effect of the SSRIs is characterized by a dose–response relationship, low doses being superior to placebo but inferior to the optimal ones; above these low doses there was however no support for the highest doses being the most effective. Second, the ES for the difference between placebo and an optimal SSRI dose is of a more respectable magnitude than has usually been attributed to the antidepressant effect of the SSRIs, hence casting doubt on the claim that these drugs are devoid of clinically relevant antidepressant properties. Third, a small but significant superiority of SSRIs over placebo in reducing *depressed mood* is observed already after 1 week of treatment.

## Figures and Tables

**Table 1:** Included trials and baseline characteristics of the study population

*Trial*	*Center (s)*	*Study conducted*	*% Female*	*DM baseline*		*HDRS-17 baseline*		*Doses and randomization*	*Observations*					
				*Mean*	*s.d.*	*Mean*	*s.d.*		*Week 1*	*Week 2*	*Week 3*	*Week 4*	*Week 5*	*Week 6*
GSK/009	10	1985–1986	50	2.8	0.6	22.6	3.1	Placebo, PRX IR 10, 20, 30 and 40 mg randomized 1:2:2:2:2	416	372	338	307	23^a^	259
GSK/274	1	1983–1988	72	2.5	0.6	23.4	3.1	Placebo and PRX IR 30 mg randomized 1:1	36	32	3^a^	29	N/A	N/A
GSK/276	1	1982–1984	50	2.5	0.7	25.1	5.8	Placebo and PRX IR 30 mg randomized 1:1	32	32	1^a^	26	0^a^	15
GSK/279^b^	2	1983–1986	69	2.1	0.8	20.9	3.5	Placebo and PRX IR 30 mg randomized 1:1	26	16	5^a^	11	10	9
GSK/810	40	2001–2002	59	2.8	0.5	23.5	3.1	Placebo, PRX CR 12.5 and 25.0 mg randomized 1:1:1	418	403	385	367	44^a^	319
GSK/874	46	2003–2004	61	2.8	0.6	22.8	3.8	Placebo, PRX CR 12.5 and 25.0 mg randomized 1:1:1	479	466	7^a^	396	36^a^	0^a^
PZ/101	11	1982–1983	34	3.1	0.8	26.6	5.9	Placebo, SER 50, 100, 200 and 400 mg randomized 1:1:1:1:1	118	92	78	66	N/A	N/A
PZ/103	7	1984–1985	53	3.0	0.6	25.1	3.0	Placebo, SER 50, 100 and 200 mg randomized 1:1:1:1	324	281	246	222	190	191
PZ/310	16	1982–1983	72	2.8	0.8	24.4	5.4	Placebo, SER 50, 100, 200 and 400 mg randomized 1:1:1:1:1	170	151	139	130	N/A	N/A
LB/89303	18	1989–1990	70	2.8	0.7	23.7	6.3	Placebo, CIT 20 and 40 mg randomized 1:1:1	193	0^a^	171	156	0^a^	146
LB/91206	12	1992–1993	60	2.9	0.5	21.9	3.2	Placebo, CIT 10, 20, 40 and 60 mg randomized 1:1:1:1:1	587	545	512	478	442	439

Abbreviations: CIT, citalopram; CR, controlled-release; DM, depressed mood; GSK, GlaxoSmithKline; HDRS-17, Hamilton Depression Rating Scale 17-item version; IR, immediate-release; LB, Lundbeck; N/A, not applicable (4 week study); PRX, paroxetine; PZ, Pfizer; SER, sertraline.

a No assessment according to the study protocol

.

b The ethics committee prohibited the use of a placebo-control for one of the participating centers.

**Table 2:** Categorical outcomes at week 6

*Group*	*Response*				*Remission*			
	*%*		*Odds ratio (CI)*	P	*%*		*Odds ratio (CI)*	P
*HDRS-17*								
Placebo versus low-dose	44.7	51.6	1.32 (1.01–1.72)	0.04	24.8	33.5	1.53 (1.13–2.06)	0.005
Placebo versus optimal-dose	44.7	61.6	1.98 (1.53–2.57)	<0.001	24.8	39.9	2.01 (1.51–2.68)	<0.001
Low-dose versus optimal-dose	51.6	61.6	1.51 (1.19–1.90)	<0.001	33.5	39.9	1.32 (1.03–1.68)	0.03
*Depressed mood*								
Placebo versus low-dose	70.5	78.8	1.56 (1.15–2.13)	0.005	15.6	27.9	2.09 (1.47–2.96)	<0.001
Placebo versus optimal-dose	70.5	85.9	2.56 (1.86–3.50)	<0.001	15.6	35.0	2.91 (2.08–4.06)	<0.001
Low-dose versus optimal-dose	78.8	85.9	1.64 (1.21–2.22)	0.002	27.9	35.0	1.39 (1.08–1.80)	0.01

Abbreviation: CI, confidence interval; HDRS-17, Hamilton Depression Rating Scale 17-item version.
